# More fuel to the fire: some patients with non-celiac self-reported wheat sensitivity exhibit adaptive immunological responses in duodenal mucosa

**DOI:** 10.1186/s12876-020-01564-w

**Published:** 2020-12-09

**Authors:** Antonia Isabel Castillo-Rodal, Janette Furuzawa-Carballeda, Mario Peláez-Luna, José Castro-Gómez, Yolanda López-Vidal, Luis Uscanga

**Affiliations:** 1 Department of Microbiology and Parasitology, Facultad de Medicina, Universidad Nacional Autónoma de México, Alcaldía de Coyoacán, Mexico City, Mexico; 2 Department of Immunology and Rheumatology, Instituto Nacional de Ciencias Médicas y Nutrición Salvador Zubirán, Vasco de Quiroga 15, Alcaldía de Tlalpan, 14000 Mexico City, Mexico; 3 Department of Gastroenterology, Instituto Nacional de Ciencias Médicas y Nutrición Salvador Zubirán, Vasco de Quiroga 15, Alcaldía de Tlalpan, 14000 Mexico City, Mexico

**Keywords:** Celiac disease, Non-celiac gluten sensitivity, Non-celiac wheat sensitivity, Intraepithelial lymphocytes, T cells, Cytokines

## Abstract

**Background:**

In contrast to the well-characterized Celiac Disease (CD), the clinical scenarios encompassed by the non-celiac self-reported wheat sensitivity (NCSRWS) might be related to different antigens that trigger distinct immune-inflammatory reactions. Although an increased number of intestinal intraepithelial lymphocytes is observed at the inception of both diseases, the subsequent immunopathogenic pathways seem to be different. We aimed to describe the cytokine profile observed in the duodenal mucosa of patients with NCSRWS.

**Methods:**

In a blind, cross-sectional study, we included duodenal biopsies from 15 consecutive untreated patients with active CD, 9 individuals with NCSRWS and 10 subjects with dyspepsia without CD and food intolerances. Immunohistochemistry and flow-cytometry were used to determine the presence of pro-inflammatory cytokine expressing monocytes and monocyte-derived dendritic cells involved in innate immune activation, cytokine-driven polarization and maintenance of Th1 and Th17/Th 22, and anti-inflammatory/profibrogenic cytokines.

**Results:**

The percentage of cells expressing all tested cytokines in the lamina propria and the epithelium was higher in CD patients than in the control group. Cytokines that induce and maintain Th1 and Th17 polarization were higher in CD than in NCSRWS and controls, also were higher in NCSRWS compared to controls. Similar differences were detected in the expression of IL-4 and TGF-1, while IL-10-expressing cells were lower in NCSRWS patients than in controls and CD subjects.

**Conclusions:**

NCSRWS patients exhibit components of both, innate and adaptive immune mechanisms but to a lesser extent compared to CD.

## Background

Celiac disease (CD) and non-celiac gluten sensitivity (NCGS) are gluten related disorders (GRD) that share clinical characteristics but have marked serological and histological differences. While autoantibodies and duodenal villus atrophy (VA) are mandatory to diagnose CD, they must be absent in order to establish a presumptive diagnosis of NCGS [1–3].

Due to the lack of specific biomarkers, in patients with normal duodenal biopsies and negative CD serology, the diagnosis of NCGS is largely based upon complex and seldom performed clinical evaluations such as double-blind gluten-placebo challenge in which symptoms improve during a gluten-free diet and symptoms relapse once gluten ingestion is resumed. In these cases, wheat allergy should also be ruled out [4–6].

CD is a well-characterized disorder with specific histological and serological features that affects genetically predisposed individuals. The ingestion of gluten and related proteins triggers well known immunopathogenic mechanisms orchestrated by CD4+ T helper 1 (Th1) and Th17 cells that result in mucosal inflammation and VA [7–12]. In contrast, NCGS is a poorly characterized disorder in which the role of gluten as the main antigen and the pathophysiologic mechanisms responsible for tissue damage and symptoms development is debatable [13].

Many subjects claiming to be intolerant to gluten were unable to associate their symptoms with the ingestion of gluten after being exposed to a double-blind gluten-placebo challenge, suggesting that other factors or mechanisms such as fermentable short-chain carbohydrates (FODMAP) or amylase-trypsin inhibitors (ATI), a group of proteins present in wheat that induce an immune-inflammatory reaction in the duodenal mucosa, may be responsible for the symptoms rather than gluten itself [14–18].

It seems plausible that most non-celiac self-reported gluten intolerant (NCSRGI) subjects are in fact wheat intolerant rather than gluten intolerant, thus some authors have suggested the term non-celiac wheat sensitivity (NCWS) instead of NCGS [19]. Although NCWS includes other potential components present in wheat, it seems to exclude the pathogenic role of other cereals.

The exhibited response to FODMAP ingestion by subjects with NCWS resembles that of food intolerance, with no evidence of an inflammatory component or histopathological alterations that could explain the reported symptoms. In contrast, the immune mediated reaction that follows the exposure to ATI could explain the symptoms and histopathological alterations observed in some patients with non-celiac self-reported wheat sensitivity (NCSRWS) [18, 20].

Thus, NCSRWS may encompass patients with food intolerance symptoms induced by FODMAP, patients with a real gluten sensitivity that triggers an immune inflammatory reaction mediated by innate immunity and patients in whom the exposure to ATI triggers an innate immune response. In all cases, certain components of adaptive immunity may also be involved [21–23].

In this exploratory study we aimed to describe the cytokine profile and quantify the pro-inflammatory cytokine-expressing monocyte and monocyte-derived dendritic cells involved in innate immune activation, cytokine-driven polarization and maintenance of Th1 polarization and Th17/Th22, and anti-inflammatory/profibrogenic cytokines in the duodenal mucosa of a group of subjects with self-reported wheat sensitivity.

## Methods

### Patients and controls

In this blind, cross-sectional study we included 15 consecutive untreated patients with active CD and 9 individuals with NCSRWS who attended from 2014 to 2016 to the outpatient gastroenterology clinic at the Instituto Nacional de Ciencias Médicas y Nutrición Salvador Zubirán, a tertiary referral medical facility in Mexico City. CD was diagnosed when patients met the following criteria: (1) CD compatible clinical data: chronic diarrhea, weight loss, bloating, abdominal discomfort, fatigue or nutrient deficiencies, (2) positive IgA anti endomysium antibodies (EMA IgA; IF, Inova Diagnostics, San Diego, CA, USA. Normal < 1:5), IgA tissue Transglutaminase antibodies (anti-tTg IgA; ELISA, Orgentec; Mainz, Germany. Normal < 10 U/mL) and IgA/IgG anti deaminated gliadin peptide antibodies (IgA and IgG-DGP; ELISA, Orgentec; Mainz,Germany. Normal < 10 U/mL) and (3) duodenal mucosa VA according to Marsh–Oberhuber criteria [24].

The diagnosis of NCSRWS was considered when patients presented with (1) intestinal and extra intestinal symptoms associated with the ingestion of gluten-containing food, (2) a clear clinical response while they were on a gluten-free diet, (3) relapse of symptoms with the ingestion of gluten-containing food, (4) negative CD serological markers (EMA IgA, anti-tTG IgA, anti IgA/IgG -DGP), (5) no evidence of wheat allergy (IgE serological test) and, (6) normal duodenal mucosa.

A qualified nutritionist with expertise in CD evaluated all patients. Symptom severity (abdominal discomfort or pain, bloating, diarrhea, and constipation) was assessed at baseline, while on an unrestricted diet, 6 weeks after following a gluten-free diet and after completing a 6 weeks challenge with 10 g of gluten per day using a visual analog scale (VAS; 0–10). Only NCSRWS patients underwent a gluten free/gluten containing diet challenge. We did not perform a double-blind gluten/placebo-controlled trial challenge in any case.

Diet compliance was evaluated during bi-weekly out-patient visits. All serological tests (EMA IgA, anti-tTG IgA, anti-DGP) were performed at baseline visit and after completion of a gluten free diet or gluten challenge.

Headache, tingling or numbness in feet or hands, fatigue, musculoskeletal pain, brain fog (mild transient cognitive impairment), rash and oral ulcers were considered extra-intestinal symptoms and they were specifically evaluated using a visual analog scale (VAS; 0–10). A good clinical response to the gluten-free diet was considered when there was a decrease in the intensity of symptoms of at least 50% compared to the baseline VAS score.

We excluded patients with other gastrointestinal diseases, history of gastrointestinal surgery, active or previous infection diseases, clotting disorders, renal insufficiency, pregnancy or breast feeding, active use of antimicrobial, probiotics, immunosuppressive drugs, non-steroidal anti-inflammatory drugs or corticosteroids. The 10 subjects included in the control group had undergone an upper endoscopy, fulfilled the functional dyspepsia ROMA III criteria and had both: negative CD serology and normal duodenal histology [25].

### Biopsy sampling

All endoscopic duodenal biopsies were obtained while the patient was on a gluten containing diet. During upper endoscopy four tissue samples from the second portion of the duodenum were obtained; two of them were placed immediately in ice-chilled Hank buffer solution (HBSS)/5% fetal bovine serum (SFB, GIBCO). The others were fixed in 10% formaldehyde and subsequently embedded in paraffin wax and cut into 4 μm thick slices.

### Isolation of mucosal lymphocytes (mLs) (IELs) from duodenal tissue

Mucosa samples (epithelium and lamina propria) were cut with a scalpel blade and incubated in phosphate buffer 1× (PBS)/ethylenediamine tetra acetic acid (EDTA) 2 mM at 34 °C for 30 min while being agitated. After that, samples were treated with Collagenase IV (Sigma) at 60 U/mL for 1 h at 34 °C while being agitated. The cell suspension was then passed through a 40 μm cell strainer (Cell Strainer BD Falcon), washed with 2 ml of PBS, and centrifuged at 800*g* for 10 min at 25 °C. The resulting pellet was homogenized in 1 mL of PBS and incubated with 1 μL of Brefeldin A (BD Golgi Plug) for 1 h at 37 °C with 5% CO_2_. Live-dead assay and cellular count from cellular samples was performed (> 90%) on a Neubauer chamber (trypan blue) as previously reported [26].

### Immunohistochemistry

Tissues placed on positively charged slides were incubated with mouse monoclonal anti-human IL-1, IL-6, IL-8, IL-10, IL-15, IL-22, IL-23, IFN-**γ**, TNF-**α**, and with rabbit polyclonal anti-human IL-2, IL-12p40, IL-17A, IL-21, or TGF-beta1 antibody (Abcam, Cambridge, MA, USA) or anti-human IL-4 antibody (Bio Legend Inc., San Diego, CA, USA) at 10 µg/mL during 30 min. Binding was detected with Universal Dako labelled streptavidin biotin reagent + peroxidase for primary antibodies from rabbit, mouse and goat (Dako, Glostrup, Denmark). Spleen and ganglion samples were used as a positive control. Negative controls were carried out with normal human serum (1:100) and with the immunohistochemistry universal negative control reagent (Enzo Life Sciences, Inc., Farmingdale, NY, USA), while phosphate buffer saline-egg albumin (SIGMA-Aldrich) was use in the reactive blank. Controls excluded nonspecific staining or endogenous enzymatic activities. We examined three different sections of each biopsy. As we have done before, cytokine-expressing cells were reported as the percentage of positive cells in three fields (X320) taken from the epithelium and lamina propria [27]. Results are expressed as the median, mean and 5th/95th percentiles.

### Peripheral blood mononuclear cells (PBMCs) isolation

Using a sample of venous blood, we isolated PBMCs by gradient centrifugation on Ficoll-Paque (Merck-Millipore). The bottom was resuspended in 1 mL of PBS 1×/Brefeldin A (BD GolgiPlug) and incubated at 37 °C in 5% CO_2_ during 1 h. Live-dead assay (trypan blue) and cellular count were performed on cellular samples (> 90%).

### Flow cytometry

1X10 PBMCs or mLs were labeled with 5 μL of antihuman CD4-FITC-labeled, monoclonal antibody (BioLegend San Diego, CA). Cells were permeabilized with 200 μL of cyto10×/cytoperm solution (BD Biosciences). Intracellular staining was performed with an anti-human Foxp3-PE-, IFN-**γ**-APC-Cy7-, IL-17A- PE-Cy7- (BioLegend), T-bet-PerCP-Cy5.5- (BD Pharmingen, San Jose, CA), and ROR-**γ**t- APC-labeled (R&D Systems, Minneapolis, MN) mouse monoclonal antibodies.

From the electronic bi-parametric gate of the singlets and living cells, we performed an analysis in the CD4+ lymphocytes population to identify CD4+/Foxp3+ cells, CD4+/T-bet cells, CD4+/INF-**γ**cells, CD4+/ROR-**γ**t+ cells, CD4+/IL-17A cells.

Results are expressed as the relative percentage of CD4+/IL-17A+-, CD4+/IFN-**γ**+-, CD4+/Foxp3+-, CD4+/T-bet+-, and CD4+/ROR-**γ**t + expressing cells in each gate. For an autofluorescence control, we ran an unstained and permeabilized cell sample. An AbC anti-mouse bead kit (Invitrogen, UK) was used to adjust instrument settings, to set fluorescence compensation, and to check instrument sensitivity. Fluorescence minus one (FMO) control were stained in parallel.

As in prior reports from our group samples were analyzed with an Attune Acoustic Focusing Cytometer Blue/Red (Life Technologies) [26, 28]. We recorded more than 10,000 events for each sample, and they were analyzed with Attune® Cytometric Software v2.1

### Ethical considerations

This work was performed according to the principles expressed in the Declaration of Helsinki. The study was reviewed and approved by the institutional ethics and research committee (GAS-1298-14/15-1; August 11, 2014). Each patient gave and signed a written informed consent.

### Statistical analysis

Due to the exploratory nature of the study and prevalence of CD, NCSWS and FD, a convenience sampling method was used. Statistical analysis was performed using GraphPad Prism for Windows (version 6.01 GraphPad software Inc. USA).

Immunohistochemical data are expressed as the median, mean and 5/95 percentiles. We used Kruskall Wallis test for non-parametric variables. We performed one-way analysis of variance on ranks by Holm-Sidak method and Dunn’s test for all pairwise multiple comparison procedures and comparisons versus a control group. *P* < 0.05 was considered statistically significant.

## Results

Demographic and clinical characteristics of patients are summarized in Tables 1 and 2. Diarrhea, abdominal pain and bloating were the most frequent symptoms in both, CD and NCSRWS patients.

**Table 1:** Main clinical characteristics of patients with celiac disease (CD), non-celiac self-reported wheat sensitivity (NCSRWS) and control

	CD (n = 15)	NCSRWS (n = 9)	Control (n = 10)
Age (years)			
Mean ± SD	55.2 ± 15.9	49.8 ± 13.6	53.2 ± 9.1
Female	13	9	5
Food allergies	2^a^	1^b^	0
Autoimmune disease	12	2	4
Osteopenia/osteoporosis	5	3	1
Body mass index kg/m^2^			
Mean ± SD	23.4 (21–39)	21.5 (18–25)	25 (22–28)

^a^Fish

^b^Berries

**Table 2:** Serological markers (antibodies values are expressed as median with min–max) and the main laboratory variables (chemistries are expressed as the number of patients with abnormal values)

	CD (n = 15)	NCSRWS (n = 9)	Control (n = 10)
DGP IgA (U/mL)	51 (12.3–132.3)	4.4 (3.5–19.6)	5.5 (4.2–36.6)
DGP IgG (U/mL)	32 (4–143)	6.2 (3.9–63.1)	4.2 (3.2–9.5)
tTg IgA (U/mL)	17 (2.3–436.1)	3.1 (0.6–15)	2.5 (1.5–4.9)
EmA IgA positive	11 (73%)	0	0
HLA DQ2/DQ8 positive	12^a^ (100%)	6^b^ (75%)	NA
Hemoglobin < 13.0 g/dL	5 (33%)	0	0
Ferritin < 11 ng/mL	3 (20%)	2 (22%)	0
Vitamin B12 < 180 pg/mL	1 (6%)	1 (11%)	NA
Folates < 5.9 ng/mL	6 (40%)	1 (11%)	NA
Vitamin D < 29 ng/mL	11 (73%)	3 (33%)	NA
Vitamin D < 20 ng/mL	5 (33%)	2 (22%)	NA
Albumin < 3.5 g/L	1 (6%)	1 (11%)	0 (0.0)

*DGP IgA* IgA anti-deamidated gliadin antibodies, *DGP IgG* IgG anti-deamidated gliadin antibodies, *tTg IgA* IgA anti*-*transglutaminase antibodies, *EmA IgA* IgA anti*-*endomysium antibodies, *NA* non-available

^a^HLA performed in 12 patients

^b^HLA performed in 8 patients

All NCSRWS patients completed the gluten challenge and experienced bloating and abdominal pain; one of them presented oral itching during the period of gluten ingestion. In all cases symptoms improved with a gluten-free diet.

Anti-DGP values were above the upper limit of normal in one NCSRWS case (DGP IgG 63 U/mL) and one control (DGP IgA 36 U/mL), but none of them showed alterations neither in histology nor antibodies (tTG, EMA).

### Percentage of peripheral CD4+ T cell subpopulations in PBMCs

**Fig. 1:**
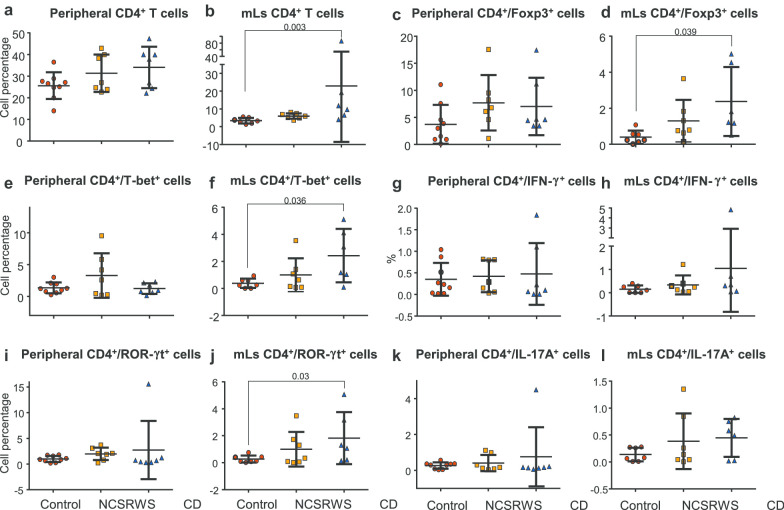
Circulating and mucosal lymphocytes (mLs) in control, non-celiac self-reported wheat sensitive (NCSRWS) and celiac disease (CD) groups. **a** Peripheral and **b** mLs CD4+ T cells; **c** Peripheral and **d** mLs CD4+/Foxp3+ Tregs; **e** Peripheral and **f** mLs CD4+/T-bet+ cells; **g** Peripheral and **h** mLs CD4+/IFN-γ+ cells; **i** Peripheral and **j** mLs CD4+/ROR-γt+ cells; **k** Peripheral and **l** mLsCD4+/IL-17A+ cells. Results are expressed as mean (black line) ± standard deviation. Control: n = 9, NCSRWS: n = 7 and CD: n = 7

No differences were observed in the number of CD4+ T cells (Fig. 1a), CD4+/Foxp3+ (Fig. 1c), CD4+/T-bet+ (Fig. 1e), CD4+/IFN**γ**-+ (Fig. 1g), CD4+/ROR-**γ**t+ neither CD4+/IL-17A+ cells (Fig. 1K) amongst the groups.

### Percentage of mucosal subpopulations in duodenal tissue

The percentage of CD4+/mLs was higher in CD patients compared to control group (*p* = 0.003, Fig. 1b). No differences were found between CD patients and NCSRWS or between NCSRWS and the control group.

CD patients had a significantly higher CD4/FoxP3 percentage in duodenum compared to the control group (*p* = 0.039, Fig. 1d). The NCSRWS CD4/FoxP3 percentage was similar to both, CD and control group.

T-bet and ROR-γt+ were higher in CD patients versus control group (*p* = 0.036, Fig. 1f and *p* = 0.03, Fig. 1j, respectively). No differences were observed between CD patients and NCSRWS. Neither there was any difference when comparing NCSRWS to the control group. The percentage of IFN-**γ**+ and IL-17A-expressing CD4 mucosal T cells in the CD group, although higher than NCSRWS, was not statistically significant amongst the three groups (Fig. 1h, l).

### Pro-inflammatory cytokines in duodenal tissue

**Fig. 2:**
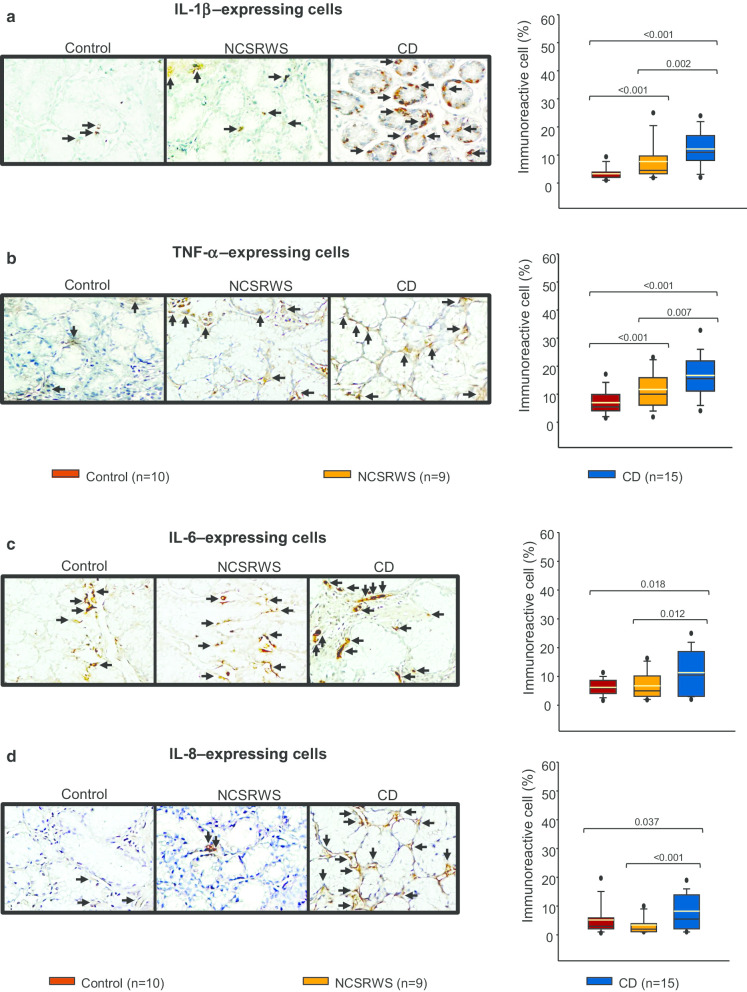
Pro-inflammatory cytokine-expressing cells in Celiac Disease. **a**–**d** left panel: Representative immunoperoxidase photomicrographs of control, non-celiac self-reported wheat sensitivity (NCSRWS) and celiac disease (CD). Arrows depict **a** IL-1β, **b** TNF-α, **c** IL-6 and **d** IL-8 immunoreactive cells. Original magnification was × 320. **a**–**d**, right panel: Relative percentage expression of **a** IL-1β, **b** TNF-α, **c** IL-6 and **d** IL-8. Results are expressed as the mean (yellow line), median (black line), and 5th/95th percentiles

The percentage of IL-beta and TNF-**α-**expressing cells in tissue of CD and NCSRWS patients was significantly higher compared to the control group. Tissue from NCSRWS patients had statistically significant lower levels of IL-1beta and TNF-**α**-expressing cells compared with CD patients (Fig. 2a, b).

The number of IL-6 and IL-8-producing cells was significantly higher in CD patients compared with control group and NCSRWS patients. No differences in the number of IL-6+ or IL-8+ cells were found in the NCSRWS group compared to the control group (Fig. 2c, d).

### Cytokines involved in the differentiation and maintenance of Th1 in duodenal tissue

**Fig. 3:**
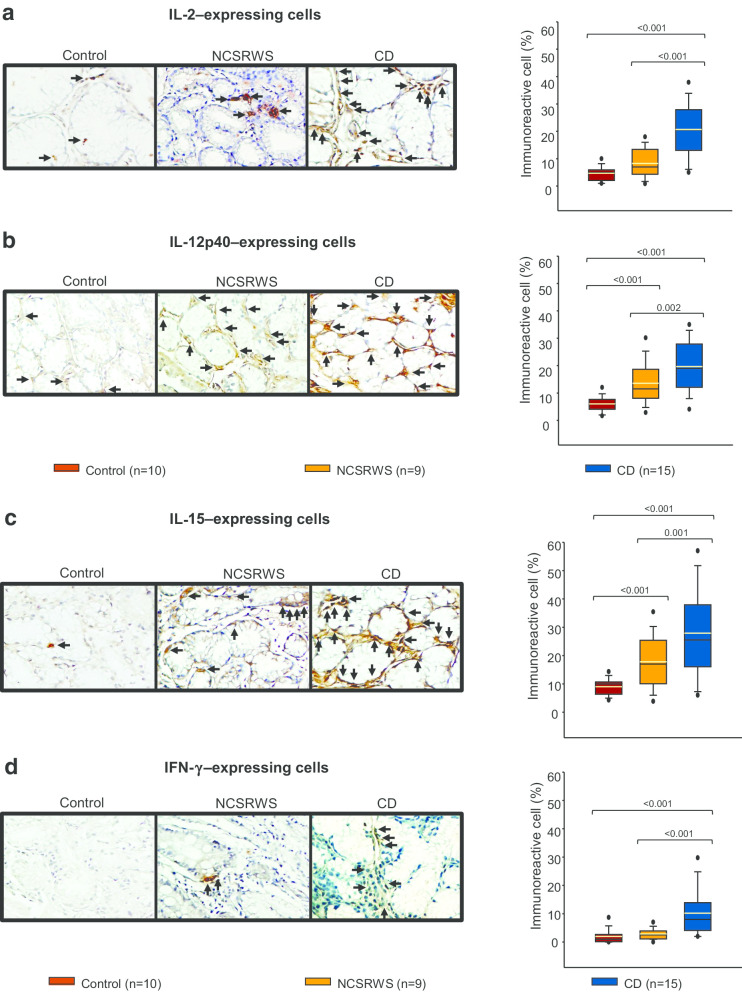
Cytokines that induce and maintain Th1 polarization. **a**–**d** left panel: Representative immunoperoxidase photomicrographs of control, non-celiac self-reported wheat sensitivity (NCSRWS) and celiac disease (CD). Arrows depict **a** IL-2, **b** IL-12p40, **c** IL-15 and **d** IFN-γ immunoreactive cells. Original magnification was × 320. **a**–**d**, right panel: Relative percentage expression of **a** IL-2, **b** IL-12p40, **c** IL-15 and **d** IFN-γ. Results are expressed as the mean (yellow line), median (black line), and 5th/95th percentiles

The percentage of IL-2+ and IFN-**γ**+ cells of CD patients was higher compared to NCSRWS and control group. No statistically significant differences in the number of IL-2+ or IFN-**γ**+ cells were determined in the NCSRWS group compared to the control group (Fig. 3a, d).

The IL-12 and IL-15 cell percentage of CD patients was conspicuously higher when compared to the control group and NCSRWS. The number of IL-12 and IL-15 expressing cells was significant higher in NCSRWS compared to controls (Fig. 3b, c).

### Cytokines involved in the differentiation and maintenance of Th17/Th22 in duodenal tissue

**Fig. 4:**
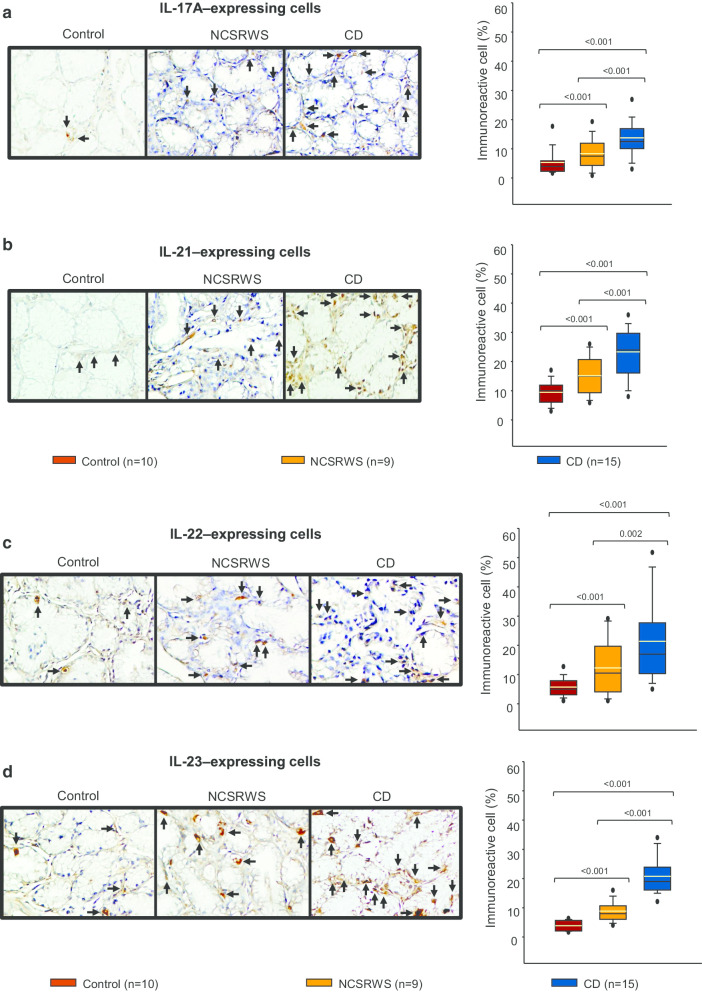
Cytokines that induce and maintain Th17 polarization. **a**–**d** left panel: Representative immunoperoxidase photomicrographs of control, non-celiac self-reported wheat sensitivity (NCSRWS) and celiac disease (CD). Arrows depict **a** IL-17A, **b** IL-21, **c** IL-22 and **d** IL-23 immunoreactive cells. Original magnification was × 320. **a**–**d**, right panel: Relative percentage expression of **a** IL-17A, **b** IL-21, **c** IL-22 and **d** IL-23. Results are expressed as the mean (yellow line), median (black line), and 5th/95th percentiles

The percentage of IL-17A+, IL-21+, IL-22+ and IL-23+ cells of CD patients was higher versus the control group, and NCSRWS patients (Fig. 4a–d). Tissue of NCSRWS group had statistically significant lower levels of IL-17A, IL-21, IL-22 and IL-23+ expressing cells compared with CD patients (Fig. 4a–d).

### Anti-inflammatory/profibrogenic cytokine expression in duodenal

**Fig. 5:**
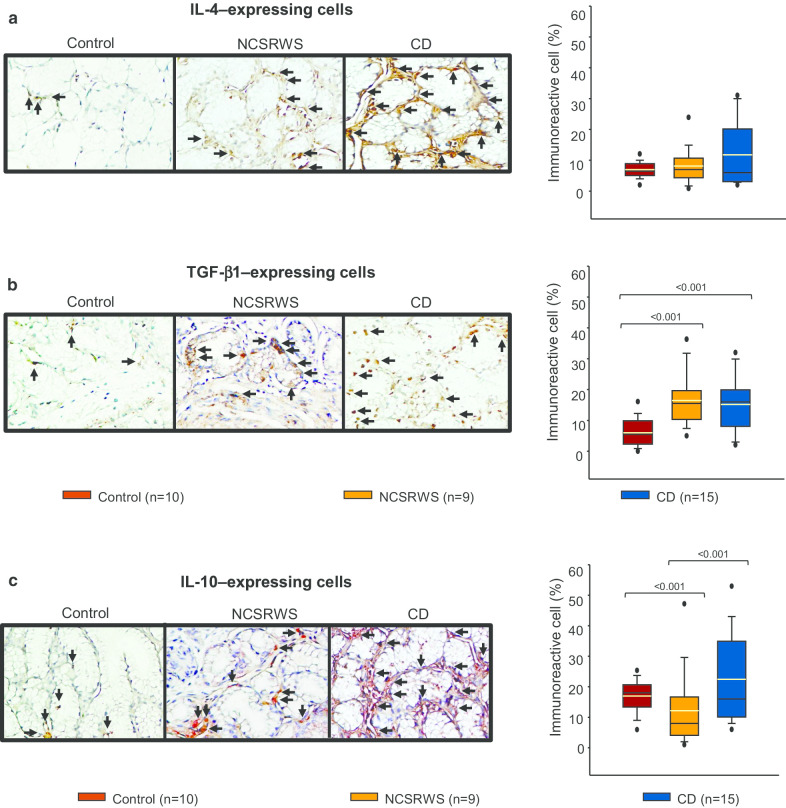
Anti-inflammatory/pro-fibrogenic cytokines. **a**–**d**, left panel: Representative immunoperoxidase photomicrographs of control, non-celiac self-reported wheat sensitivity (NCSRWS) and celiac disease (CD). Arrows depict **a** IL-4, **b** TGF-β1, and **c** IL-10 immunoreactive cells. Original magnification was × 320. **a**–**d**, right panel: Relative percentage expression of **a** IL-4, **b** TGF-β1, and **c** IL-10. Results are expressed as the mean (yellow line), median (black line), and 5th/95th percentiles

No differences were observed in IL-4 cell percentage when compared CD patients or NCSRWS versus the control group (Fig. 5a). TGF-beta1 and IL-10 expressing cells from CD or NCSRWS patients were higher versus the control group (Fig. 5b, c). No statistically significant difference was found between CD and NCSRWS patients.

## Discussion

CD is a well-characterized disease with specific histological and serological features and established immuno-pathological mechanisms that are triggered by the ingestion of gluten and related proteins in genetically predisposed individuals [9–12]. In contrast, NCGS is a disorder seeking its own identity; it is a condition that encompasses different clinical scenarios including subjects with irritable bowel syndrome and patients with food intolerances that experience abdominal or extra-intestinal symptoms after consumption of gluten. NCGS patients typically improve with a gluten-free diet [19, 29, 30]. The ambiguity about its existence is based on the absence of specific diagnostic biomarkers or histological characteristics. Its diagnosis has relied on the clinical response during complicated double-blind gluten-placebo controlled challenges that have used different vehicles and doses of gluten [5, 14, 31]. In an attempt to standardize NCGS diagnosis, a group of experts met in Salerno proposed a double-blind placebo-controlled challenge using 8 g of gluten administered over 2 periods of one week each and separated by one-week wash-up term [6]. Since the Salerno criteria is difficult to fulfill in the clinical setting, once CD and wheat allergy have been reasonably ruled out, the presumptive diagnosis of NCGS is based merely on the clinical response to a diet with and without gluten. However, even with the use of double-blind gluten-placebo controlled challenge a large number of self-defined gluten intolerant subjects fail to relate their symptoms to the ingestion of gluten which suggests that other wheat components, such as FODMAP and ATI, rather than gluten itself, may play a central pathological role [13–18]. Despite these diagnostic caveats, we are confident that our self-considered gluten intolerant patients are whole wheat sensitive/intolerant considering that all of them improved while following a gluten-free diet and relapsed when they were exposed to a gluten-containing diet. On the other hand, CD and wheat allergy were reasonable excluded based on serological test and histological features.

Since we did not perform a double-blind gluten-placebo-controlled challenge the participation of other antigens that are present in wheat, besides gluten, cannot be ruled out. Nevertheless, in daily clinical practice these patients are classified as NCGS subjects.

Flow cytometry from samples obtained from all cases during an unrestricted diet showed that CD4+ cell subpopulations were quite different. As expected, CD patients exhibited a wide range of innate and adaptive immune responses when compared to the control group. The percentage of inflammatory and regulatory cells CD4+T, CD4+/Foxp3+, CD4+/T-bet+, CD4+/ROR-**γ**t+ were higher in CD than in the control group and NCSRWS patients. Importantly, the percentage of inflammatory and regulatory CD4+ cells was higher in the group of NCSRWS patients when compared to the control group but lower than that observed in CD subjects. Although these comparisons were not finding statistically significant, they resemble a previous report and suggest that an inflammatory process is present in these self-reported wheat intolerant subjects [19].

Immunohistochemical analysis showed the most noticeable changes. To our knowledge, this is the first description of cytokine production on duodenal mucosa from NCSRWS patients using immunohistochemistry.

The percentage of pro-inflammatory cytokine-expressing cells in the duodenal mucosa was higher in patients than in controls. The inflammatory response was conspicuously higher in CD subjects although pro-inflammatory cytokine- expressing cells were also evident in NCSRWS patients, except for IL-6- and IL-8-expressing cells. These findings have been previously reported by other studies that evaluated cytokine levels on serum and peripheral blood mononuclear cells or duodenal mucosa culture supernatants by ELISA [32–36].

Present evidence suggests that innate immune response plays a central role in the pathophysiology of NCGS. Increased expression of toll-like receptor 2 (TLR-2) and 4 (TLR-4), claudin 4 (CLD-4), and TNF-**α** has been shown by different groups in subjects with self-reported gluten intolerance [37]. According to this concept, IL1 and TNF-**α**-expressing cells were higher in NCSRWS patients compared to the control group. These findings support the widely demonstrated participation of innate immunity in both, CD and NCGS [10, 11, 14, 35]. Since our objective was to determine the cytokine expression in CD and NCSRWS patients we did not consider necessary to evaluate TLRs. It should be noted that our results pertaining IL-6 and IL-8 in NCSRWS patients are similar to those reported in mononuclear cells culture supernatants (ELISA) from NCGS subjects [32]. On the other hand, we determined the presence of IL-6+ and IL-8+ cells using tissue immunohistochemistry, but we did not determine the individual cellular cytokine production.

Th cell polarization from naïve precursors is a tightly controlled process where IL-12 and IL-15 play a central role as factors involved in the differentiation of Th1 response [38, 39].The secreted IL-2 by activated antigen-specific CD4+ and CD8+ T cells, is consumed at the same and distant sites by cells expressing the IL-2R (effector T cells, NK cells and Tregs). IL-2 acts via STAT5; it influences the differentiation of Th1, Th2 and Th17 cell subsets, and maintains the transcriptional program for Treg function [40]. In addition, IL-2 probably stimulates the differentiation of other cell groups while IL-12 does it for Th1 and, IL-22 and IL-23 for Th17. In contrast, IL-15 not only promotes the increase of IEL in CD but also supports Th1 and Th17 response [41–43]. Meeting these concepts, we observed that the percentage of cytokine expressing cells that induce and maintain Th1 and Th17 polarization in the mucosa of CD patients was higher compared to the other groups. Interestingly, IL-2 and IL-12 expressing cells were also increased in NCSRWS group compared to the control group, suggesting the participation of some adaptive immunity components in these self-reported wheat intolerant subjects. However, If the main effect of IL-17 observed in our work is on innate or adaptive immune response remains unclear. IL-17 is produced by CD4+ T lymphocytes, innate immune cells (e.g., such as T cells, neutrophils, macrophages, NK, NKT cells) and by non-immune cells including epithelial and parenchymal cells, which means that it constitutes the first line of the host defense and acts even before the adaptive immunity could be initiated [44, 45].

Recent evidence supports the participation of adaptive immune mechanisms in NCGS patients. High levels of IFN-**γ** have been found in the duodenal and rectal mucosa of NCGS patients that have completed a gluten challenge [46, 47].

Moreover, it has been shown that these subjects are able to produce specific antibodies against native gliadins and that about 50% of them express the high-risk haplotypes HLA-DQ2/DQ8 for CD [48]. It should be noted that we found these CD’s high risk haplotypes in 6 out of 8 patients with NCSRWS and in all CD subjects.

The number of cells expressing TGF-beta1 was notably high in both, CD and NCSRWS patients. It is mostly certain that this represents an anti-inflammatory response elicited by peptides derived whether from prolamins in the case of CD cases or from other antigens in NCSRWS patients [18, 20]. Remarkably, IL-10 expressing cells were not increased in CD or in NCSRWS; actually, they were lower in NCSRWS than in the other groups.

Although our findings in CD patients resemble prior reports some differences in our wheat intolerant patients can be noted. Our patients with NCSRWS were diagnosed on clinical bases excluding wheat allergy and CD, however, we did not perform a double-blind gluten-placebo challenge in any case and the participation of other antigens, besides gluten, could not be excluded. It could be argued that in fact, these might be patients with seronegative CD considering that the majority presented with high-risk haplotype (HLA DQ2/DQ8 genes), however all of them showed a normal duodenal mucosa while being on a gluten-containing diet [48].

Due to these diagnostic limitations regarding NCGS, these patients represent a true challenge in clinical practice. At present time it seems clear that other wheat components like FODMAP or ATI are capable to trigger symptoms and immune-inflammatory reactions in some non-celiac self-reported wheat intolerant subjects.

## Conclusions

Non-celiac self-reported wheat sensitive subjects show components of both, innate and adaptive immunity response in the duodenal mucosa. We believe that our results provide one more piece in the complicated puzzle of wheat sensitivity.

## Data Availability

The data sets during or analyzed during the current study available from the corresponding author on reasonable request.

## Abbreviations

CD: Celiac disease
NCGS: Non-celiac gluten sensitivity
NCSRWS: Non-celiac self-reported wheat sensitivity
GT-FD: Gluten tolerant patients with functional dyspepsia
IL-4: Interleukin 4
TGF- 1: Tumor growth factor 1
IL 10: Interleukin 10
HLA: Human leukocyte antigen
GRD: Gluten related disorders
VA: Villous atrophy
Th1: T helper 1 lymphocytes
Th17: T helper 17 lymphocytes
Th22: T helper 22 lymphocytes
EmA IgA: Anti-endomysium antibodies
Anti-tTg IgA: Anti-transglutaminase antibodies
AGA-DGP IgA and IgG: Anti-deamidated gliadin peptide antibodies
GFD: Gluten free diet
VAS: Visual analog scale
FD: Functional dyspepsia
HBSS: Hank buffer solution
SFB: Fetal bovine serum
IEL: Intraepithelial lymphocytes
mLs: Mucosal lymphocytes
PBS: Phosphate buffer
EDTA: Ethylenediamine tetra acetic acid
IL-1: Interleukin 1
IL-6: Interleukin 6
IL-8: Interleukin 8
IL-15: Interleukin 15
IL-22: Interleukin 22
IL-23: Interleukin 23
IFN: Interferon
TNF: Tumor necrosis factor
IL-2: Interleukin 2
IL-12p40: Interleukin 12p40
IL-17A: Interleukin 17A
IL-21: Interleukin 21
PBMC: Peripheral blood mononuclear cells
ULN: Upper limit of normal
IBS: Irritable bowel syndrome
TLR-2: Toll-like receptor 2
TLR-4: Toll-like receptor 4
CLD-4: Claudin 4
NK: Natural killer cells
Treg: T regulatory cells
FODMAP: Fermentable short-chain carbohydrates
ATI: Amylase-trypsin inhibitors

## References

[1] Gasbarrini GB, Mangiola F (2014). Wheat related disorders:A broadspectrum of evolving diseases. United Eur Gastroenterol J.

[2] Catassi C, Bai JC, Bonaz B (2013). Non-celiac gluten sensitivity: the new frontier of gluten related disorders. Nutrients.

[3] Czaja-Bulsa G (2015). Non coeliac gluten sensitivity—a new disease with gluten intolerance. Clin Nutr.

[4] Holmes G (2013). Non coeliac gluten sensitivity. Gastroenterol Hepatol Bed Bench.

[5] Carroccio A, Mansueto P, Iacono G (2012). Non-celiac wheat sensitivity diagnosed by double-blind placebo-controlled challenge: exploring a new clinical entity. Am J Gastroenterol.

[6] Catassi C, Elli L, Bonaz B (2015). Diagnosis of non-celiac gluten sensitivity (NCGS): the Salerno expert´s criteria. Nutrients.

[7] Meresse B, Ripoche J, Heyman M, Cerf-Bensussan N (2008). Celiac disease: from oral tolerance to intestinal inflammation, autoimmunity and lymphomagenesis. Nat Muc Immunol.

[8] Schuppan D, Junker Y, Barisani D (2009). Celiac disease: from pathogenesis to novel therapies. Gastroenterology.

[9] Kupfer SS, Jabri B (2012). Pathophysiology of celiac disease. Gastrointest Endosc Clin N Am.

[10] Fasano A, Catassi C (2012). Clinical practice. Celiac disease. N Engl J Med.

[11] Stamnaes J, Sollid LM (2015). Celiac disease: autoimmunity in response to food antigen. Semin Immunol.

[12] Caio G, Volta U, Sapone A (2019). Celiac disease: a comprehensive current review. BMC Med.

[13] Nijeboer P, Bontkes HJ, Mulder CJ, Bouma G (2013). Non-celiac gluten sensitivity. Is it the Gluten or the Grain?. J Gastrointestin Liver Dis.

[14] Molina-Infante J, Carroccio A (2017). Suspected non-celiac gluten sensitivity confirmed in few patients after gluten challenge in double-blind, placebo-controlled trials. Clin Gastroenterol Hepatol.

[15] Biesiekierski JR, Peters SL, Newnham ED, Rosella O, Muir J, Gibson PR (2013). No effects of gluten in patients with self-reported non-celiac gluten sensitivity after dietary reduction of fermentable, poor-absorbed short-chain carbohydrates. Gastroenterology.

[16] Zanini B, Baschè R, Ferraresi A (2015). Randomized clinical study: gluten challenge induces symptom recurrence in only a minority of patients who meet clinical criteria for non-celiac gluten sensitivity. Aliment Pharmacol Ther.

[17] Skoje GI, Sarna VK, Minelle H (2018). Fructan, rather than gluten, induces symptoms in patients with self-reported non-celiac gluten sensitivity. Gastroenterology.

[18] Schuppan D, Zevallos V (2015). Wheat amylase trypsin inhibitors as nutritional activators of innate immunity. Dig Dis.

[19] Mansueto P, Soresi M, Iacobucci R (2019). Non-celiac wheatsensitivity: a search for the pathogenesisof a self-reported condition. Italian J Med.

[20] Zevallos VF, Raker V, Tenzer S (2017). Nutritional wheat amylase-trypsin inhibitors promote intestinal inflammation via activation of myeloid cells. Gastroenterology.

[21] Sapone A, Lammer KM, Mazzarella G (2010). Differential mucosal IL-17 expression in two gliadin-induced disorders: gluten sensitivity and the autoimmune enteropathy celiac disease. Int Arch Immunol.

[22] Sapone A, Lammers KM, Casolaro V (2011). Divergence of gut permeability and mucosal gene expression in two gluten associated conditions: celiac disease and gluten sensitivity. BMC Med.

[23] Molina-Infante J, Santolaria S, Montoro M, Esteve M, Fernández-Bañares F (2014). Non-celiac gluten sensitivity: a critical review of current evidence. Gastroenterol Hepatol.

[24] Oberhuber G, Granditsch G, Vogelsang H (1999). The histopathology of coeliac disease: time for a standardized report scheme for pathologists. Eur J Gastroenterol Hepatol.

[25] Ford AC, Bercik P, Morgan DG, Bolino C, Pintos-Sanchez MI, Moayyedi P (2014). The Rome III criteria for the diagnosis of functional dyspepsia in secondary care are not superior to previous definitions. Gastroenterology.

[26] Valle J, Morgado JMT, Ruíz-Martín J (2017). Flow cytometryofduodenal intraepithelial lymphocytesimproves diagnosis ofceliac diseasein difficultcases. United Eur Gastroenterol J.

[27] Furuzawa Carballeda J, Fonseca Camarillo G, Yamamoto-Furusho JK (2016). Interleukin 27 is upregulated in patients with active inflammatory bowel disease. Immunol Res.

[28] Leon F (2011). Flow cytometry of intestinal intraepithelial lymphocytes in celiac disease. J Immunol Methods.

[29] Mansueto P, D’Alcamo A, Seidita A, Carroccio A (2015). Food allergy inirritablebowel syndrome: the case of non-celiacwheat sensitivity. World J Gastroenterol.

[30] Vazquez-Roque MI, Camilleri M, Smyrk T (2013). A controlled trial of gluten free diet in patients with irritable bowel syndrome-diarrhea: effects on bowel frequency and intestinal function. Gastroenterology.

[31] Figueroa-Salcido OG, Ontiveros N, Cabrera-Chavez F (2019). Glutenvehicle andplacebo for non-celiac sensitivity assessment. Medicina (Kaunas).

[32] Masaebi F, Azizmohammad Looha M, Rostami-Nejad M, Pourhoseingholi MA, Mohseni N, Samasca G, Lupan I, Rezaei-Tavirani M, Zali MR (2020). The predictive value of serum cytokines for distinguishing celiac disease from non-celiac gluten sensitivity and healthy subjects. Iran Biomed J.

[33] Heydari F, Rostami-Nejad M, Moheb-Alian A, Mollahoseini MH, Rostami K, Pourhoseingholi MA, Aghamohammadi E, Zali MR (2018). Serum cytokines profile in treated celiac disease compared with non-celiac gluten sensitivity and control: a marker for differentiation. J Gastrointest Liver Dis.

[34] Ido H, Matsubara H, Kuroda M, Takahashi A, Kojima Y, Koikeda S, Sasaki M (2018). Combination of gluten-digesting enzymes improved symptoms of non-celiac gluten sensitivity: a randomized single-blind, placebo-controlled crossover study. Clin Transl Gastroenterol.

[35] Di Sabatino A, Giuffrida P, Fornasa G, Salvatore C, Vanoli A, Naviglio S, De Leo L, Pasini A, De Amici M, Alvisi C, Not T, Rescigno M, Corazza GR (2016). Innate and adaptive immunity in self-reported nonceliac gluten sensitivity versus celiac disease. Dig Liver Dis.

[36] Brottveit M, Beitnes AC, Tollefsen S, Bratlie JE, Jahnsen FL, Johansen FE, Sollid LM, Lundin KE (2013). Mucosal cytokine response after short-term gluten challenge in celiac disease and non-celiac gluten sensitivity. Am J Gastroenterol.

[37] Junker Y, Ziessig S, Kim SJ (2012). Wheatamylase trypsin inhibitorsdrive intestinalinflammation via activationoftoll-likereceptor 4. J Exp Med.

[38] Zhou W, Zhang F, Aune TM (2003). Either IL-2 or IL-12 is sufficient to direct Th1 differentiation by nonobese Diabetic T Cells. J Immunol.

[39] Feili-Hariri M, Falkner DH, Morel PA (2005). Polarization of naive T cells into Th1 or Th2 by distinct cytokine-driven murine dendritic cell populations: implications for immunotherapy. J Leukoc Biol.

[40] Bosman O, Sprent J (2012). The role of interleukin-2 during homeostasis and activation of the immune system. Nat Rev Immunol.

[41] Mention JJ, Ben Ahmed M, Beque B (2003). Interleukin 15: a key to disrupted intraepithelial lymphocyte homeostasis and lymphomagenesis in celiac disease. Gastroenterology.

[42] Harris KM, Fasano A, Mann DL (2008). Monocytes differentiated with IL-15 support Th17 and Th1 responses to wheat gliadin: implications for celiac disease. Clin Immunol.

[43] Ross SH, Cantrell DA (2018). Signaling and function of Interleukin-2 in T Lymphocytes. Annu Rev Immunol.

[44] Chong DLW, Ingram RJ, Lowther DE, Muir R, Sriskandan S, Altmann D (2012). The nature of innate and adaptive interleukin-17A responses in sham or bacterial inoculation. Immunology.

[45] Solaymani-Mohammadi S, Eckmann L, Singer SM (2019). Interleukin (IL-21) in inflammation and immunity during parasitic diseases. Front Cell Infect Microbiol.

[46] Di Liberto D, Mansueto P, D’Alcamo A (2016). Predominance oftype1 innate lymphoidcells in the rectal mucosa of patients with non-celiacwheat sensitivity: reversal aftera wheat-free diet. Clin Transl Gastroenterol.

[47] Volta U, Bardela MT, Calabro A, Troncone R, Corazza GR (2014). Study group for non-celiac gluten sensitivity. An Italian prospective multicenter survey on patients suspected of having non-celiac gluten sensitivity. BMC Med.

[48] Schepatti A, Sanders DS, Biagi F (2018). Seronegative coeliac disease: clearing the diagnostic dilemma. Curr Opin Gastroenterol.

